# Orthopaedic Disorders in Myotonic Dystrophy Type 1: descriptive clinical study of 21 patients

**DOI:** 10.1186/1471-2474-14-338

**Published:** 2013-12-01

**Authors:** Lisa Schilling, Raimund Forst, Jürgen Forst, Albert Fujak

**Affiliations:** 1 Department of Orthopaedic Surgery, Friedrich-Alexander-Universität Erlangen-Nürnberg, Rathsberger Str. 57, Erlangen D-91054, Germany

**Keywords:** Myotonic dystrophy type 1, Curschmann-steinert disease, Orthopaedic disorders, Spinal deformities, Foot deformities, Contractures, Fractures, Orthopaedic treatment, Cardiac involvement, Pulmonary involvement

## Abstract

**Background:**

Myotonic Dystrophy Type 1 (DM1) is the most common form of hereditary myopathy presenting in adults. This autosomal-dominant systemic disorder is caused by a CTG repeat, demonstrating various symptoms. A mild, classic and congenital form can be distinguished. Often the quality of life is reduced by orthopaedic problems, such as muscle weakness, contractures, foot or spinal deformities, which limit patients’ mobility.

The aim of our study was to gather information about the orthopaedic impairments in patients with DM1 in order to improve the medical care of patients, affected by this rare disease.

**Methods:**

A retrospective clinical study was carried out including 21 patients (11 male and 10 female), all diagnosed with DM1 by genetic testing. All patients were seen during our special consultations for neuromuscular diseases, during which patients were interviewed and examined. We also reviewed surgery reports of our hospitalized patients.

**Results:**

We observed several *orthopaedic impairments*: *spinal deformities* (scoliosis, hyperkyphosis, rigid spine), *contractures* (of the upper extremities and the lower extremities), *foot deformities* (equinus deformity, club foot, pes cavus, pes planovalgus, pes cavovarus, claw toes) and *fractures*.

Five patients were affected by *pulmonary diseases* (obstructive airway diseases, restrictive lung dysfunctions). Twelve patients were affected by *cardiac disorders* (congenital heart defects, valvular heart defects, conduction disturbances, pulmonary hypertension, cardiomyopathy).

Our patients received *conservative therapy* (physiotherapy, logopaedic therapy, ergotherapy) and we prescribed *orthopaedic technical devices* (orthopaedic custom-made shoes, insoles, lower and upper leg orthoses, wheelchair, Rehab Buggy). We performed *surgery* for spinal and foot deformities: the scoliosis of one patient was stabilized and seven patients underwent surgery for correction of foot deformities.

**Conclusions:**

An orthopaedic involvement in DM1 patients should not be underestimated. The most common orthopaedic impairments are contractures, foot deformities and spinal deformities. Contractures are typically located distally in the lower extremities, but can also occur in the hip or shoulder joints. Foot deformities could be treated with orthopaedic custom-made shoes, orthoses or insoles. Surgery is indicated for severe foot deformities or contractures.

## Background

Myotonic Dystrophy Type 1 (DM1) or Curschmann-Steinert disease is the most common form of hereditary myopathy presenting in adults [1]. This autosomal-dominant systemic disorder is caused by a CTG repeat in the DMPK (myotonic dystrophy protein kinase) gene on chromosome 19q13.3 [2]. More than 35 repeats lead to a toxic gain of function causing abnormal alternative splicing, a premature termination and finally truncated, inactive muscle-specific chloride channels (CIC-1) as well as a defective calcium reuptake pump of the sarcoplasmatic reticulum (SERCA1) [3,4]. People with a premutation (under 50 repeats) do not have symptoms, they only carry the risk of passing on larger repeat sizes to their children [3]. Patients with up to 150 repeats have the mild form of DM1, phenotypically presenting with cataracts, mild myotonia and normal life expectancy [3]. The classic form (up to 1,000 repeats) starts between 10 and 30 years of age [3] with symptoms, such as distal muscle weakness, facial dysplasia, cardiac conduction defects [5,6], gastrointestinal manifestations [3], cataracts [7], cognitive changes and more rarely, skin diseases and endocrine dysfunctions [3]. Furthermore, the risk of pregnancy complications is elevated. The average age of death ranges from 48 to 55 years [3]. The more severe congenital form of DM1 is usually characterised by more than 2,000 CTG repeats, an age of onset between birth and 10 years of age and a shortened lifespan of approximately 45 years [3]. The first symptoms occur during pregnancy, such as reduced fetal movements and polyhydramnios [3]. Patients suffer from the classic symptoms as well as from infantile hypotonia, positional malformations [8], respiratory problems [9] and mental handicap [3].

The quality of life is often reduced by orthopaedic problems, such as muscle weakness, contractures, foot or spinal deformities limiting patients’ mobility. That makes it difficult for patients to work or take part in daily activities and social life [10].

The aim of our study was to gather information about the orthopaedic disorders in patients with DM1 who visited our special consultations for neuromuscular diseases in order to improve the medical care of patients, affected by this rare disease.

## Methods

The data for this study was collected during our special consultations for patients with neuromuscular diseases, during which patients were interviewed and examined. We also reviewed surgery reports of our hospitalized patients.

In our study we included 21 patients, 11 male and 10 female, diagnosed with DM1. In all cases the diagnosis was confirmed by genetic testing. Seventeen patients (81%) suffered from the severe congenital form and four (19%) from the adult onset form of DM1. Eighteen (86%) had a positive family history of DM1, see Table 1.

**Table 1:** Patient population and follow-up data

**Patient**	**Gender**	**Visits**	**Follow-up (y)**	**Age at first visit**	**Age at latest visit**	**Family history**
1	m	14	10,49	4,62	15,12	+
2	f	2	0,98	19,66	20,64	+
3	f	7	2,95	15,91	18,86	+
4	m	1	0	45,48	45,48	-
5	f	4	1,96	46,59	48,55	+
6	m	25	14,09	0,48	14,57	+
7	f	4	3,68	15,05	18,73	+
8	f	6	2,6	1,3	3,88	+
9	f	8	5,35	7,42	12,77	+
10	m	2	5,08	1,71	6,79	-
11	m	5	3,97	4,14	8,11	+
12	m	12	5,89	0,33	6,22	+
13	m	2	0,5	4,44	4,94	+
14	m	3	0,86	16,2	17,02	+
15	f	26	8,73	0,87	9,6	+
16	m	2	0,98	5,19	6,17	+
17	m	2	0,98	2,67	3,64	+
18	f	3	0,17	18,16	18,87	+
19	f	11	1,24	0,23	1,47	+
20	f	1	0	62,15	62,15	+
21	m	1	0	48,58	4,58	-

The average age of our patients was 15.3 (±18.9; 0.2 - 62.2) years at their first visit and 18,7 (±17.4; 1.47 - 62.2) years at their latest visit, which measures up to a mean follow-up of 3.4 (±3.8; 0–14.1) years. The mean number of times patients visited our hospital was 6.7 (±7.3; 1–26). For details of patient population and follow-up data see Table 1.

This descriptive clinical study with retrospective data collection was conducted in accordance with the ethical standards for human research of the Research Ethics Committee, Faculty of Medicine, Friedrich-Alexander-Universität Erlangen-Nürnberg.

## Results

### Orthopaedic disorders

#### Spinal deformities

In our study scoliosis affected 3 patients (14%; patients 3, 7, 14). The severe scoliosis of patient 3 was left-convex with a Cobb angle of 100° (Th8-L1-L5), 45° pelvic tilt to the left. Patient 7 had a right-convex lumbar scoliosis of 10°, without pelvic obliquity. Patient 14 suffered from a right-convex scoliosis with a Cobb angle of 45° (Th11-L1-L3), 15° pelvic tilt to the right. Six patients (29%; patients 1, 2, 9, 12, 15, 16) presented with hyperkyphosis. Two patients (10%; patients 3, 14) suffered from a rigid spine with pathological chin-sternum distance. Patient 3 had a mild form with a 5 cm distance in inclination and 20 cm in reclination, whereas patient 14 had a more severe manifestation with a 10 cm distance in inclination and 26 cm in reclination.

#### Contractures

Eleven of our patients (52%) suffered from contractures. Two patients (10%; patients 18, 21) presented with contractures of the upper extremities. The external rotation of both shoulders was limited by 10° in patient 21. An extensor inhibition of the elbow joints occurred in patient 18 (10° limitation on the right side, 5° on the left side) and in patient 21 (10° on both sides).

Ten patients (48%) were affected by contractures of the lower extremities. Patient 3 had flexion contractures of the hip joints (20° on the right and 15° on the left side). In two patients (10%; patients 9, 12) the physiological hyperextensibility of the hip joints was lost, but none of our patients suffered rotation, abduction or adduction contractures of the hip joints. Patient 9 had flexion contractures of both knee joints of 5°. Six patients (29%; patients 3, 7, 10, 13, 17, 20) had a limited dorsiflexion capacity of the ankle joint (possible dorsiflexion of 10° or less). The average dorsiflexion capacity was 8° (±3°, 5° - 10°). Three patients were affected on both feet (patients 7, 10, 20), the others only on one foot (patients 3, 13, 17). A loss of dorsiflexion capacity (0° of dorsiflexion possible) occurred in eight patients (38%). Four were affected on both feet (patients 4, 5, 8, 21), four only on one foot (patients 1, 3, 16, 17). Ten patients (48%; patients 1, 6, 9, 11, 12, 14, 15, 16, 18, 19) suffered from equinus deformity (less than 0° possible dorsiflexion in the ankle joints), club foot or pes cavovarus with equinus components with an average equinus of 20° (±19°, 5° - 60°).

#### Foot deformities

In 20 out of 21 patients (95%) foot deformities occurred. With a total number of six patients (29%) equinus deformity was the most common. Five patients were affected on both feet, one patient on one foot. They had an average equinus of 19° (±17°, 5° - 60°). Three patients (14%) suffered from club feet, two of them were affected on both feet, one only on one foot. The average equinus component in club feet was 26° (±23°, 5° - 60°). Patient 3 had a club foot deformity with a limited dorsifelxion of the left ankle joint (5° of dorsiflexion). Pes planovalgus occurred in four patients (19%), pes cavus in three patients (14%) and pes cavovarus in two patients (10%). Aside from that, three patients (14%) complained of claw toes on both feet. For details of all patients’ foot deformities see Table 2.

**Table 2:** Foot deformities and treatment

**Patient**	**Deformity right foot**	**Treatment**	**Deformity left foot**	**Treatment**
**1**	Pes cavovarus	O	Pes cavovarus	O
		A (7 years)		A, T, S (7 years)
		A, T (12 years)		
**2**	No deformity		No deformity	
**3**	Pes planovalgus et adductus	O, L	Club foot	O, L
**4**	Lost dorsiflexion	L	Lost dorsiflexion	L
**5**	Lost dorsiflexion, hallux valgus, claw toes II, III		Lost dorsiflexion, hallux valgus, claw toes II, III	
**6**	Pes cavovarus	I	Pes cavovarus	I
		A, TS, P (12 years)		A, T, S, P (11 years)
**7**	Pes planivalgus	O	Pes planovalgus	O
**8**	Pes cavus	L	Pes cavus	L
**9**	Club foot, hallux valgus	O, L	Club foot, hallux valgus	O, L
		A, E (2 years)		A, E (2years)
		A, R (13 years)		A, R (13years)
**10**	Pes cavus		Pes cavus	
**11**	Pes equinus	A (6years)	Pes equinus	A (6years)
**12**	Pes equinus	O,L	Pes equinus	O.L
		A, S, E (1 year)		A, S, E (1year)
		A, R (3 years)		
**13**	Pes planovalgus	I,L	Pes planovalgus	I,L
**14**	Pes equinus	I	Pes equinus	I
**15**	Pes equinus et cavus claw toes IV,V	O,L	Pes equinus et cavus claw toes IV,V	O,L
		A (1 year)		A (1 year)
		A, T, S (3years)		A, T, S (3years)
**16**	Lost dorsifelxion	I	Pes equinus	I
**17**	Pes planovalgus	L	Pes planovalgus, lost dorsifelxion	L
**18**	Club foot, claw toes II-V	I	Club foot, claw toes toes II-V	I
		A, T (18 years)		A, T (18years)
**19**	Pes equinus	L	Pes equinus	L
		A, P, E (1 year)		A, P, E (1 year)
**20**	Limited dorsiflexion (5°)		Limited dorsiflexion (5°)	
**21**	Pes varus, lost dorsifelxion		Pes varus, lost dorsifelxion	

Conservative treatment:

O Orthopaedic custom-made shoes.

L Lower leg orthoses.

I Orthopaedic insoles.

Surgical procedures.

A Achillotenotomy.

T Transfer of the tibialis posterior muscle to os cuneiforme laterale.

S Splitting of the plantar fascia.

P Transfer of peroneus longus muscle to peroneus brevis muscle.

E Lengthening of the flexor hallucis longus muscle.

R Release of the ankle joints’ dorsal capsule.

#### Fractures

Five patients (24%; patients 2, 4, 6, 14, 15) sustained fractures. Three fractures occurred due to falls as a result of patients’ muscular imbalance. During the course of one year patient 15 broke her distal radius as well as her lower leg and patient 4 broke his big toe after multiple falls because of his unsteady gait. Patient 2 suffered a metatarsal fracture and patient 6 a talus chip fracture. The comminuted fracture of the lower leg of patient 14 was nailed, whereas all other fractures were treated conservatively.

### Cardiac and pulmonary involvement

We focused on the orthopaedic manifestations of DM1, but several patients in our study suffered from non-orthopaedic disorders of this systemic disease. DM1 is often associated with pulmonary and cardiac involvement.

Five of our patients (24%) were affected by pulmonary diseases. Three patients (14%; patients 10, 18, 20) had an obstructive airway disease (OAD). In the case of patient 10 the OAD was caused by a stenosis of the left main bronchus and, as a newborn, by bronchiomalacia. Patient 20 suffered from chronic obstructive pulmonary disease (COPD). Two patients (10%; patients 3, 14) had a restrictive lung dysfunction with reduced vital capacity. Since the age of fifteen, patient 14 needed assisted ventilation at night, because of his severe respiratory muscle weakness. Furthermore, seven of our patients (33%; patients 1, 3, 8, 12, 15, 17, 19) needed postnatal ventilatory assistance. Patient 15 was given artificial respiration a second time, when falling ill with pneumonia at the age of six months.

Twelve patients (57%) were affected by cardiac disorders. Five patients (24%; patients 1, 8, 10, 11, 12) had congenital heart defects. Patients 8 and 12 had a patent foramen ovale (PFO), patients 1 and 11 an atrial septal defect (ASD) and patient 10 had a ventricular septal defect (VSD). The VSD of patient 10 was part of a congenital double-outlet-right-ventricle. Patient 10 also had pulmonary hypertension, as well as patient 12. Four patients (19%; patients 2, 3, 8, 12) suffered from valvular heart defects. Both, mitral valve insufficiency and tricuspid insufficiency, occurred twice. Three patients (14%; patients 1, 12, 18) had conduction disturbances. Patient 12 had a right bundle branch block (RBBB) with a QRS complex of 86 ms, patients 1 and 18 had a first degree atrioventricular block. Patient 13 presented with supraventricular tachycardia (SVT). Two patients (10%; patients 5, 20) suffered from cardiomyopathy (CMP). Because of her dilated cardiomyopathy (DCM), patient 20 had a pacemaker and an implanted defibrillator. Pericardial tamponades occurred in two patients (10%; patients 12, 17).

### Treatment

#### Conservative therapy

In order to improve their movement restrictions, 18 patients (86%) attended physiotherapy regularly. In most cases the indication was gait training, improvement of coordination skills and proprioception. Furthermore, patients received posture training and mobilisation of affected joints to improve their range of motion. Children received physiotherapy to support their motoric learning process. Patients with elevated risks learned breathing techniques to optimize their lung function, which also helps to prevent pneumonia. Six patients (29%) received logopaedic support to help them with difficulties in swallowing and dysarthria. In order to manage the activities of daily living (ADL) six patients (29%) underwent ergotherapeutic treatment.

### Orthopaedic technical devices

For stabilisation and correctional purposes, six patients (29%) were fitted with orthopaedic custom-made shoes, five patients (24%) needed insoles. Nine patients (43%) wore lower leg orthoses, two of them (10%; patients 9, 17) also had upper leg orthoses. All of the nine patients wearing lower leg othoses needed them to stabilize their gait, two of them (patients 15, 19) also had a second pair of lower leg orthoses for positioning purposes at night. Patient 9 wore positioning upper leg orthoses at night and patient 17 had upper leg orthoses to improve his gait. For details of all patients’ conservative treatment of foot deformities and/or contractures of the ankle joint see Table 2. One patient (patient 14) wore a corset because of his severe scoliosis.

Eight of our patients (38%; patients 3, 8, 10, 12, 15, 16, 17, 20) were fitted with a wheelchair or a Rehab Buggy. Four of them (patients 3, 8, 12, 15) were still able to walk short distances with a wheeled walker, a mobility aid to maintain stability while walking. Because of her deteriorating ambulatory ability patient 9 also used a wheeled walker. Four patients (19%; patients 3, 8, 12, 17) used a standing frame. Three (14%; patients 8, 15, 16) used a special bike with stabilizers to be more mobile and patient 15 also used a Swivel Walker before acquiring her ambulatory ability. Two patients (10%; patients 3, 19) needed a lifter to make transfers easier.

### Surgery

#### Spinal surgery

At the age of sixteen the lumbar scoliosis of patient 3 (Cobb angle of 100° (Th8-L1-L5) left-convex, 45° pelvic tilt to the left) was stabilized. During this operation we carried out a spinal fusion with multi-segmental spinal instrumentation from the upper thoracic spine to the sacrum for correction of pelvic obliquity. The instrumentation consisted of transverse process laminar hook claws at Th3 and Th5, double Luque wires from Th6 down to Th12, pedicle screws in L1-S1 and two transverse connectors in Th5 and Th12/L1. We made an autologous bone graft between L5 and S1, using bone resected from the processi spinosi. The outcome of this operation was a stable spine situation and sitting position with an improvement to a residual scoliosis of 46° (Cobb angle of 46° (Th7-L2-L5) left-convex , 30° pelvic tilt to the left).

**Figure 1:**
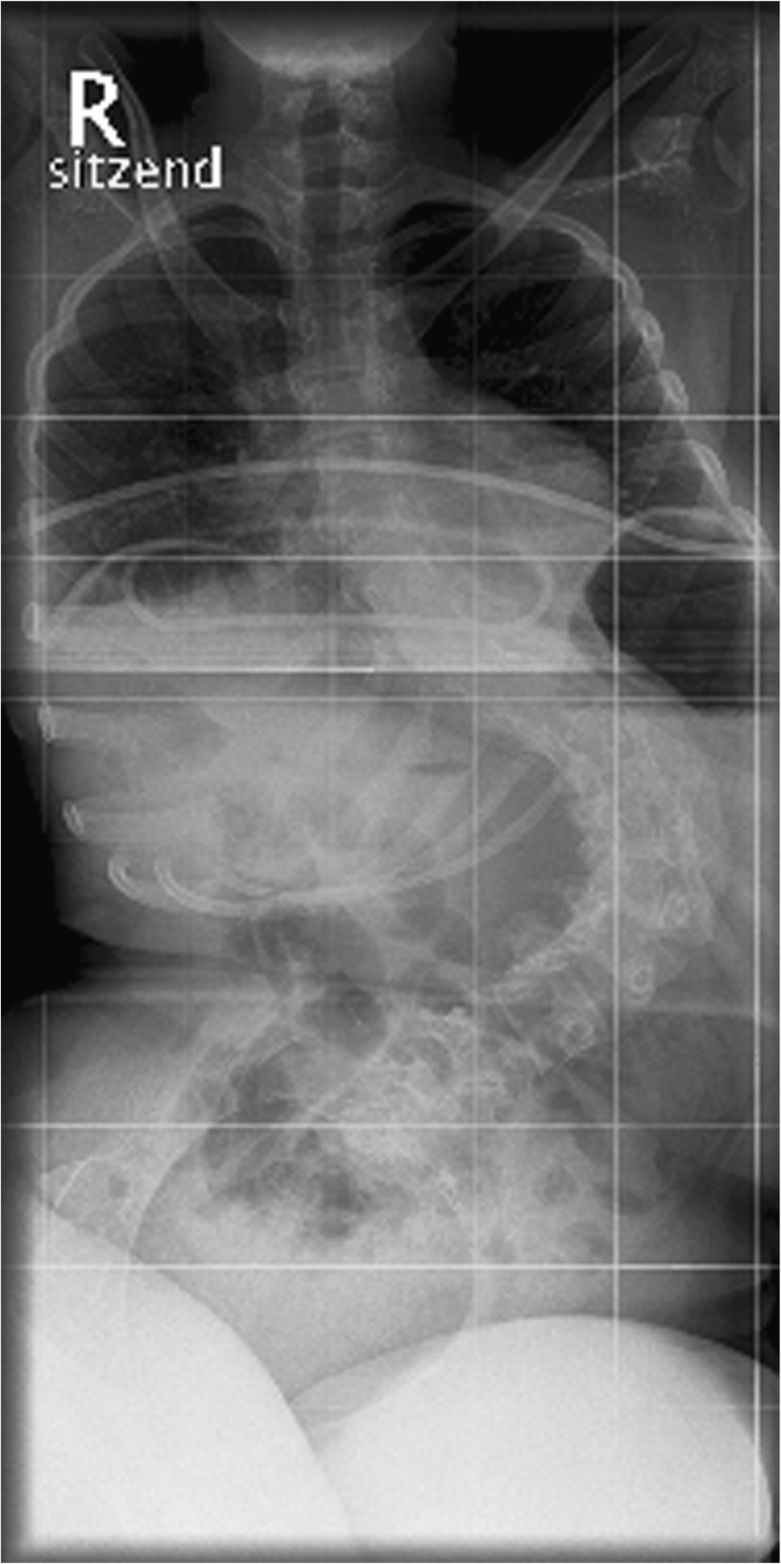
Anteroposterior preoperative X-ray of the spine: Cobb angle of 100° (Th8-L1-L5) left-convex, 45° pelvic tilt to the left.

**Figure 2:**
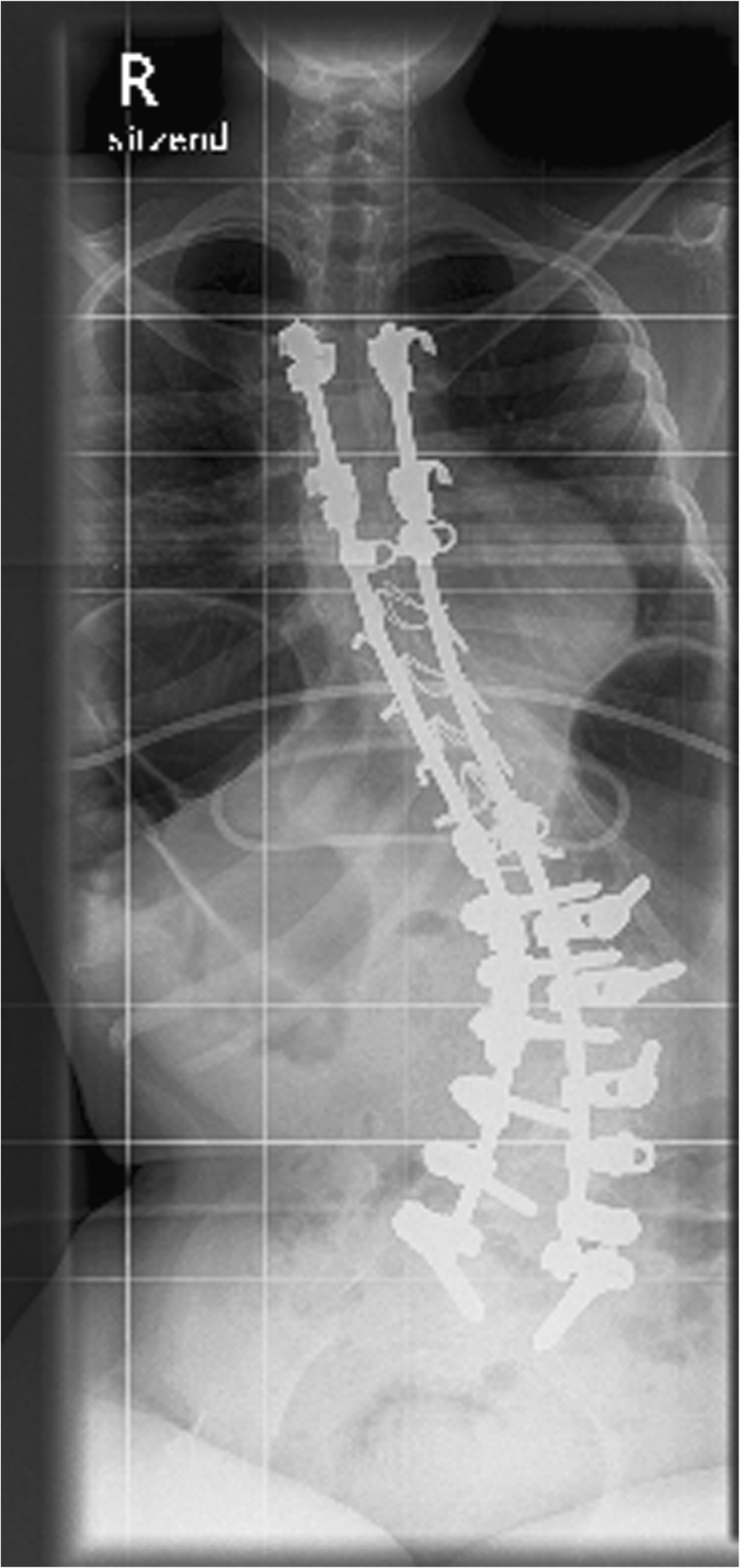
Anteroposterior X-ray of the spine at follow-up: Cobb angle of 46° (Th7-L2-L5) left-convex, 30° pelvic tilt to the left.

**Figure 3:**
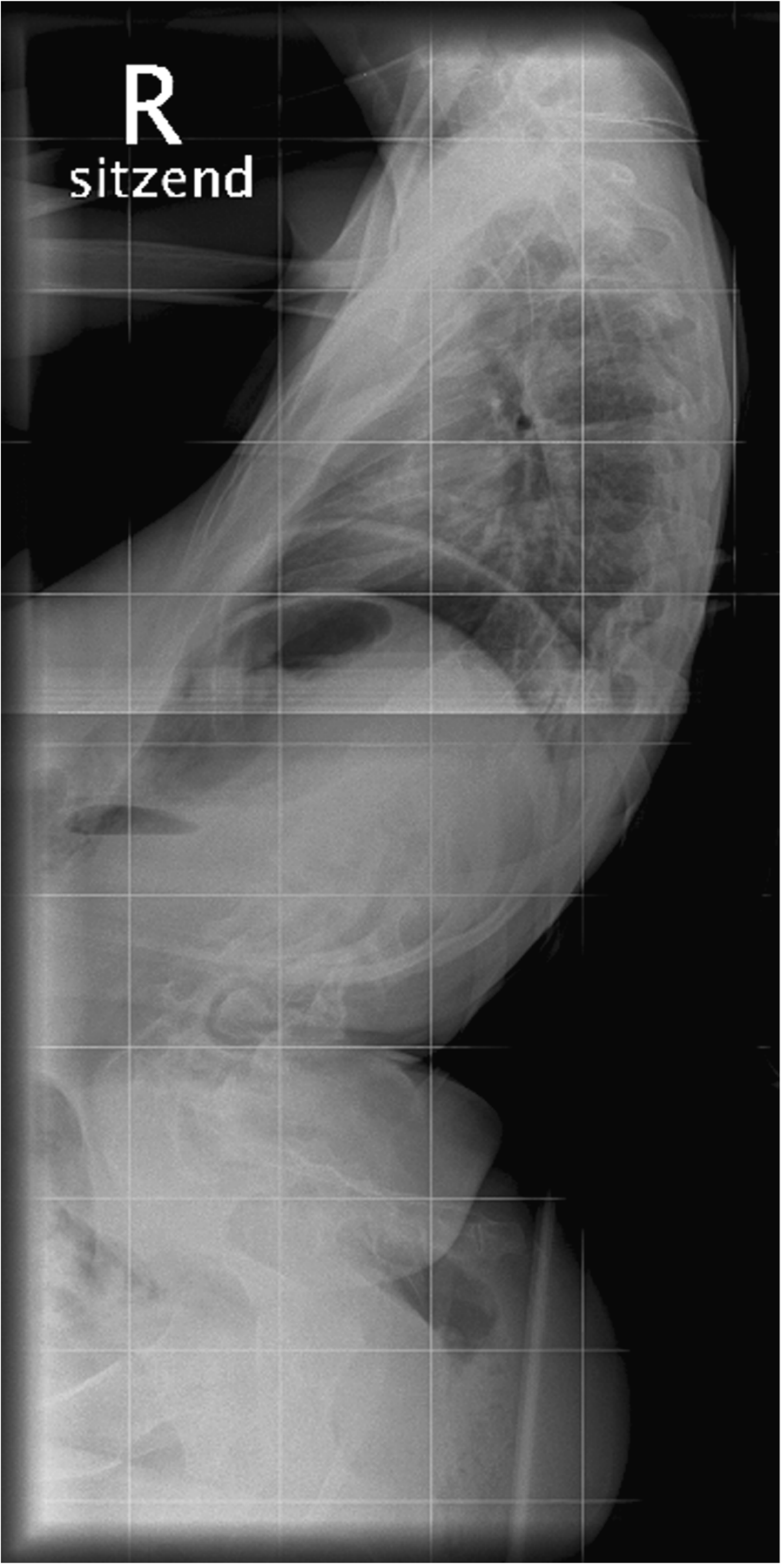
Lateral preoperative X-ray of the spine: Cobb angle of 100° (Th8-L1-L5) left-convex , 45° pelvic tilt to the left.

**Figure 4:**
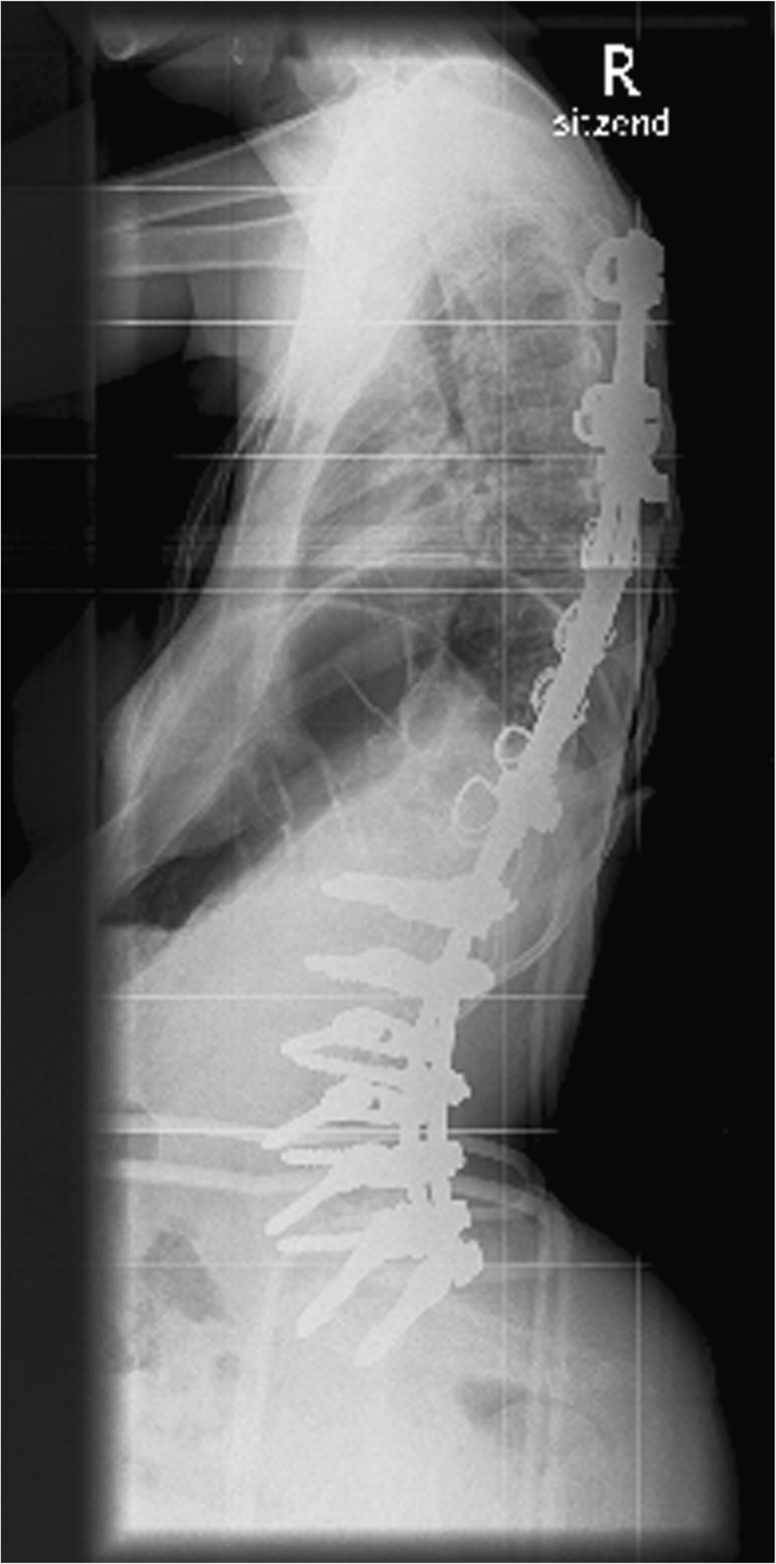
Lateral X-ray of the sp ine at follow-up: Cobb angle of 46° (Th7-L2-L5) left-convex , 30° pelvic tilt to the left.

At first, the postoperative course was without complications and patient 3 was released from hospital after three weeks, free of complaints. Two weeks later, the patient was admitted to our hospital again after discovering a wound healing disorder, when the scab dissolved. We carried out an examination of the wound and found a serous effusion (without pus) and muscle necroses in the lumbar part of the spine. We debrided the wound and removed the inserted spongiosa in toto. After irrigating the area with six litres of Jet-Lavage we inserted a sponge with vacuum system. During the next three weeks the vacuum system was changed nine times and finally, after the secondary suture, the wound healed without complications. Pre- and postoperative X-rays are provided in the attachment (Figures 1, 2, 3, 4).

#### Surgery for foot deformities and contractures of the ankle joint

Eight of the twenty patients presenting with foot deformities underwent surgery. All were treated on both feet, four of them in one and four of them in two surgical interventions. Most often the indication was pes equinus (eleven times), followed by club foot (six times) and pes cavovarus (five times). After receiving treatment for dorsiflexion contractures of the ankle joint, two contractures were cured. In fourteen cases a neutral position of the upper ankle joint was possible, but the dorsiflexion capacity was still limited. Patients could only extend the foot by an average of 7° (±3°, 5° - 10°). In six cases a neutral position but no dorsiflexion was possible. For details of treatments see Table 2.

Considering all surgical procedures for contractures of the ankle joint and/or foot deformities, complications occurred in only two patients.

At the age of 12, the club foot of patient 1 was treated with a transfer of the tibialis posterior muscle to os cuneiforme laterale and achillotenotomy. Three days after the operation we noticed that, clinically and sonografically, the tendon of the tibialis posterior muscle could not be located at the correct site of insertion. We performed revision surgery with lengthening of the tibialis posterior muscle and reinsertion. We then applied a cast to prevent another avulsion of the insertion site. We observed a positive outcome.

Patient 6 received achillotenotomy and a transfer of the tendon of the tibialis posterior muscle to os cuneiforme laterale on the right side at the age of 12. Five days after the operation, he suffered from wound dehiscence in the area of the achillotenotomy, triggered by forced mobilisation (by the non compliant patient). We irrigated the area and closed the wound by secondary suture. Twelve days after that, a second complication of the initial operation occurred. This time, the wound dehiscence was on the right medial midfoot, the area of the tendon transfer. We applied an hydrocolloid wound dressing. A week later, we removed the dressing and found a pronounced skin maceration with local flush. After opening the suture, we saw a seroma in this area. During surgery we also found a small wound dehiscence with seroma in the incision area on the distal medial lower leg. Both areas were irrigated, curetted and sutured again. A plaster splint was applied to the lower limb and a Redon-drainage was inserted in the seroma for two days. After three days of bed rest with elevation of the leg, bandage changes and lymphatic drainage, the outcome was positive.

As in both areas, in which dehiscence occurred, subcutaneous surgical suture material was used, we supposed that this patient’s reaction to the material caused the wound healing disturbance.

## Discussion

A detailed examination of orthopaedic disorders is essential to optimize therapeutic possibilities and to introduce standards for patients with DM1. Considering that, it was important to us to conduct a study focussing in detail on the orthopaedic manifestations as well as their conservative and surgical management. As the patients in this study visited our special consultations for neuromuscular diseases, the percentages of orthopaedic impairments are higher than in other studies, where patients mostly suffered from general weakness [10], cardiac abnormalities [5,6,11,12], pulmonary involvement [12] or cataract [7,13].

### Spinal deformities

The muscle weakness and hypotonia of the trunk make it more and more difficult for paediatric patients to stay in an upright position, leading to spinal deformities, most often a thoracolumbar kyphoscoliosis [14]. With 43% of orthopaedic patients affected by spinal deformities, we found that the prevalence of these disorders was higher than reported previously by Canavese and Sussman, especially the hyperkyphosis we noticed in 29% of patients has not been reported before [2]. However, since both orthopaedic studies only had a limited number of patients it is difficult to draw a general conclusion. According to Die-Smulders, it is important to treat progressive spinal deformities at an early age to be able to keep the child seated comfortably and to prevent aspiration pneumonia, one of the most frequent causes of death in patients with DM1 [15]. Daher et al., however, observed that spinal deformities in DM1 patients tend to develop slowly. They recommend to treat spinal impairments conservatively until the growth spurt in puberty, after which the spinal fusion should be performed [16]. In our study, patient 3, who gained her ambulatory ability at the age of five, was treated conservatively until the age of sixteen. Before the spinal fusion she complained of pain in the lumbar part of the spine, she was able to sit unaided and walk, holding somebody’s hand. After surgery, which improved the scoliosis and pelvic obliquity, she was pain-free and her ambulatory ability improved.

### Contractures

As DM1 mostly affects the distal muscles [3], contractures are typically located distally in the lower extremities, a fact our study confirms with ten patients (48%) suffering from equinus deformity or foot deformities with equinus components. One patient had flexion contractures of the knee joints. We also observed, however, flexion contractures of the hip joints and two cases in which the physiological hyperextensibility of the hip joints was lost. Patients also had contractures of the upper extremities, two patients were affected by contractures of the elbow joints and one patient suffered from contractures of the shoulder joints.

### Foot deformities

Concerning foot deformities, our study confirms the results of Canavese and Sussman [2], with equinus deformity being the most common (29%). In our study, the prevalence of club feet was a little lower (14%), whereas the prevalence of pes planovalgus (19%) and claw toes (14%) was higher than reported by Canavese and Sussman. Furthermore, we found a prevalence of pes cavus of 14% and of pes cavovarus of 10%. They recommended orthoses for children with equinus deformity to provide standing stability and to prevent planar flexion contractures [2]. In our study, three children with equinus deformity, but also three with planovalgus deformity, one with club feet, one with cavus deformity and one child with a limited dorsiflexion, profited from orthoses. If the deformity is not severe or patients have a good ambulatory ability, we recommend orthopaedic shoes, orthoses or insoles, which helped fifteen of our patients to improve stability, balance and gait. Transfer of the tibialis posterior muscle resulted in good varus correction, according to Griffet et al. as well as Canavese and Sussman, who recommended this surgical procedure also for equinus deformity [2,17]. Our results confirm that, with four cavovarus deformities, three equinus deformities and two club feet being successfully treated. Griffet et al. recommended achillotenotomy for better dorsiflexion after observing a group of patients, including sixteen cases of Duchenne muscular dystrophy, two cases of DM1 and one case of progressive muscular dystrophy (PMD) [17]. They found an improvement of 9° (±12°) in dorsiflexion for equinus deformity and 6° (±5°) for varus deformity [17]. After performing achillotenotomy twenty-two times in our group of DM1 patients, we observed an improvement of overall 6° (±6°, 0° - 20°) in dorsiflexion. The indications were: twenty cases of equinus deformity or other foot deformities with equinus components (improvement of 7° (±6°, 0° - 20°)) and twice lost dorsiflexion capacity (improvement of 8° (±4°, 5° - 10°). Because of these significant improvements, we recommend achillotenotomy for equinus deformity and other foot deformities with equinus components, if necessary in combination with another tendon transfer or soft tissue technique.

### Complications

According to other studies, patients with DM1 are at high risk of suffering anaesthetic complications [8,18,19]. Mathieu et al. reported that the overall frequency of these complications was 8,2%, most of them pulmonary [18]. Sinclair and Reed observed a 10% rate of respiratory complications in their group of DM1 patients [19]. We performed twenty-five surgical procedures on eight patients, requiring narcosis. Twenty-four were performed under endotracheal anaesthesia and one with laryngeal mask. We observed no anaesthetic complications, but two forms of postoperative complications. One case of an avulsion of the tendon after transfer of the tibialis posterior muscle (patient 1) and two cases of wound dehiscence (patients 3, 6). The avulsion of the tendon (patient 1) as well as the wound dehiscence of patient 6 was caused by noncompliance due to their mental handicap. In the case of patient 6, noncompliance in combination with his reaction to the subcutaneous surgical suture material caused the wound dehiscence.

## Conclusions

In conclusion, conducting a detailed clinical examination of orthopaedic disorders in DM1 patients is important, as an orthopaedic involvement should not be underestimated. The most common orthopaedic impairments are contractures as well as foot and spinal deformities.

Contractures are typically located distally in the lower extremities, but can also occur in the hip or shoulder joints.

Foot deformities should be treated with orthopaedic custom-made shoes, orthoses or insoles. If patients suffer from severe foot deformities or contractures of the ankle joint, surgery is indicated. According to our experience we can recommend transfer of the tibialis posterior muscle for correction of club foot and pes cavovarus as well as achillotenotomy to treat equinus deformity. Depending on the deformity, several soft tissue interventions can be indicated and can be performed in one operation.

## Abbreviations

ADL: Activities of daily living; ASD: Atrial septal defect; CIC-1: Chloride channel, voltage-sensitive 1; CMP: Cardiomyopathy; CTG: Cytosine-thymine-guanine (base triplet); COPD: Chronic obstructive pulmonary disease; DCM: Dilated cardiomyopathy; DM1: Myotonic dystrophy type 1; DMPK: Myotonic dystrophy protein kinase; L: Lumbar vertebrae; OAD: Obstructive airway disease; PFO: Patent foramen ovale; PMD: Progressive muscular dystrophy; RBBB: Right bundle branch block; S: Sacral vertebrae; SERCA: Sarco-/endoplasmatic reticulum Ca2 + -ATPase; SVT: Supraventricular tachycardia; Th: Thoracic vertebrae; VSD: Ventricular septal defect.

## Competing interests

The authors declare that they have no conflicts of interest.

## Authors’ contributions

AF and JF examined the patients and collected the data. LS assisted in examination of patients, collected and prepared the data. AF and RF were the clinical and academic supervisors. LS wrote the first draft. All authors were involved in the interpretation of data, preparation and revision of manuscript. All authors read and approved the final manuscript.

## Pre-publication history

The pre-publication history for this paper can be accessed here:

http://www.biomedcentral.com/1471-2474/14/338/prepub
